# Australasian Pigeon Circoviruses Demonstrate Natural Spillover Infection

**DOI:** 10.3390/v15102025

**Published:** 2023-09-29

**Authors:** Babu Kanti Nath, Tridip Das, Andrew Peters, Suman Das Gupta, Subir Sarker, Jade K. Forwood, Shane R. Raidal, Shubhagata Das

**Affiliations:** 1 School of Agricultural, Environmental and Veterinary Sciences, Faculty of Science and Health, Charles Sturt University, Wagga Wagga, NSW 2678, Australia; tdas@csu.edu.au (T.D.); apeters@csu.edu.au (A.P.); shraidal@csu.edu.au (S.R.R.); sdas@csu.edu.au (S.D.); 2 Biosecurity Research Program and Training Centre, Gulbali Institute, Charles Sturt University, Wagga Wagga, NSW 2678, Australia; sgupta@csu.edu.au (S.D.G.); jforwood@csu.edu.au (J.K.F.); 3 Biomedical Sciences & Molecular Biology, College of Public Health, Medical and Veterinary Sciences, James Cook University, Townsville, QLD 4814, Australia; subir.sarker@jcu.edu.au; 4 School of Dentistry and Medical Sciences, Faculty of Science and Health, Charles Sturt University, Wagga Wagga, NSW 2678, Australia; 5 Training Hub Promoting Regional Industry and Innovation in Virology and Epidemiology, Gulbali Institute, Charles Sturt University, Wagga Wagga, NSW 2678, Australia

**Keywords:** pigeon circovirus, PCR, phylogeny, spillover

## Abstract

Pigeon circovirus (PiCV) is considered to be genetically diverse, with a relatively small circular single-stranded DNA genome of 2 kb that encodes for a capsid protein (Cap) and a replication initiator protein (Rep). Australasia is known to be the origin of diverse species of the Order Columbiformes, but limited data on the PiCV genome sequence has hindered phylogeographic studies in this species. To fill this gap, this study was conducted to investigate PiCV in 118 characteristic samples from different birds across Australia using PCR and sequencing. Eighteen partial PiCV *Rep* sequences and one complete PiCV genome sequence were recovered from reservoir and aberrant hosts. Phylogenetic analyses revealed that PiCV circulating in Australia was scattered across three different subclades. Importantly, one subclade dominated within the PiCV sequenced from Australia and Poland, whereas other PiCV sequenced in this study were more closely related to the PiCV sequenced from China, USA and Japan. In addition, PiCV Rep sequences obtained from clinically affected plumed whistling duck, blue billed duck and Australian magpie demonstrated natural spillover of PiCV unveiled host generalist characteristics of the pigeon circovirus. These findings indicate that PiCV genomes circulating in Australia lack host adapted population structure but demonstrate natural spillover infection.

## 1. Introduction

Circoviruses have co-evolved in specified host niches for millennia including several diverse avian species, ground dwelling mammals, fish and even crustaceans [1,2,3,4,5,6]. Among the many circovirus species discovered to date, some cause chronic degenerative or immunosuppressive diseases in the reservoir host species while sustaining the ability to induce frequent spillover infections in naïve or aberrant hosts [7,8]. Pigeon circovirus (PiCV) is a pathogen in pigeons with a global distribution [9,10]. Although many studies have highlighted the immunosuppressive effects of PiCV in natural hosts and considered this to be the cause of young pigeon disease syndrome (YPDS), a recent study satisfied Henle-Koch’s postulates and confirmed pigeon rotavirus as the cause of YPDS-like diseases in domestic pigeons [11,12,13,14]. PiCV genetic diversity has been studied in Europe and China [15,16,17,18], but not in Australasia, where Columbiformes have their greatest extant diversity. Some reports from Australia suggest a high nucleotide diversity of PiCV among different species groups of pigeon, hinting towards a deep host specified divergence [19].

PiCV belongs to the Genus circovirus of the Family circoviridae, comprising of small non-enveloped viruses with single stranded circular DNA genomes of 1.7–2.5 kilobases (kb) [19]. It has two main genes which are bidirectionally transcribed from a double-stranded replicative intermediate, with the virion sense gene (Rep, ORF V1) encoding a replication-associated protein (Rep) and the complementary sense gene (cap, ORF C1) encoding a capsid protein (Cap) [20,21,22]. PiCV is considered to be a contributing agent to YPDS, a multifactorial condition characterised by lethargy, weight loss, respiratory distress, diarrhoea and poor performance in young racing pigeons aged 1 to 4 months [12,15]. Over the past 20 years, PiCV infections have been documented in feral, racing, ornamental and meat pigeons of all ages in many different parts of the world [11,18,19,23,24,25,26,27]. The virus is transmitted mainly horizontally through ingestion or inhalation of virus-contaminated faecal material and feather dust [28]. However, the detection of viral DNA in tissues of embryos, ovaries and testes of some birds suggests that PiCV is also transmitted vertically [27]. In addition to the lymphoid organs, PiCV-infected cells were also detected by PCR in the liver, kidney, trachea, lung, brain, crop, intestine, spleen, bone marrow and heart of birds [29].

Spillover infection has been most common in avian circoviruses such as beak and feather disease virus (BFDV), with recent fatalities recorded in distant hosts spanning distant Orders [7,30]. Spillover infection between distantly related Orders highlights a deeper host-codivergence with recycling of evolutionary memory via frequent spillover and host jumps over millennia [31]. Recent research on circovirus ecology and evolution has focused on spillover events of BFDV, which is one of the most ubiquitous pathogens of Australian avifauna [32]. Intra- and inter-Order spillover events have been documented for BFDV; however, not as many reports are available for PiCV, although deep genetic recombination is noted within extant PiCV alignments [17,19]. In the present study, PiCV genomes were analysed from a range of host species, primarily to appreciate host and geographically based divergence, as well as probable spillover events.

## 2. Materials and Methods

### 2.1. Sample Collection

As part of the routine diagnostic investigation for morbidity and mortality, diverse group of pigeons, doves and ducks from different geographic regions of Australia were tested for pigeon circovirus at the Charles Sturt University Veterinary Diagnostic Laboratory, Wagga Wagga, NSW, Australia. Moreover, dried blood spots from archived pigeon samples from Papua New Guinea (PNG) were also used for PiCV investigation in this study. A PiCV positive sample CS20-1270 (GenBank accession number OM470912) sourced from a rock pigeon in Australia in 2020 was used as a control for PCR testing.

### 2.2. DNA Extraction, PCR, Library Construction and Sequencing

A total of 118 genomic DNA were extracted from blood (*n* = 42), tissues (*n* = 14), feathers (*n*= 18) and cloacal swabs (*n* = 44) using protocols described elsewhere [33,34]. PCR primers were designed based on all available complete genome sequences from NCBI GenBank and using the design tools in the Geneious Prime v 2021.1.1 package. Oligonucleotide primers specifically targeting the replication associated protein coding gene (Rep) for the PiCV genome (forward, PiCV SD-F, 5′-GTGAAAGCCGGAAGAGCAATG-3′, and reverse, PiCV-SD-R, 5′-GTGATGACGATGACTTCCGTTTTGAAG-3′) were used for the PCR amplification of an approximately 139 bp fragment [35]. To obtain the 515 bp fragment of replication associated protein coding gene (Rep) for the PiCV genome, a new pair of primers (forward, PiCV-BK-F, 5′-ATGCCATTGTGGGGAAGGAG-3′, and reverse, PiCV-BK-R, 5′-GCCAGCCGTAGAAGTCATCA-3′) were used for the PCR amplification. The PCR reaction mix contained 2 μL of template genomic DNA, 25 pM of each primer (Sigma-Aldrich, St. Louis, MO, USA), 1.5 mM MgCl_2_, 1.25 mM of each dNTP, 1× GoTaq^®^ Green Flexi Reaction Buffer, 1 U of Go Taq DNA polymerase (Promega Corporation, Madison, WI, USA) and nuclease free H2O (Invitrogen, Waltham, MA, USA) to make up 25 uL for each reaction. PCR was carried out in an C1000TM thermal cycler (Bio-Rad, Hercules, CA, USA) under the following conditions: denaturation at 95 °C for 5 min, followed by 40 cycles of 95 °C for 30 s, 57 °C for 45 s and 72 °C for 2 min and a final extension step of 5 min at 72 °C. PCR amplicons were visualised in 1.5% agarose gel stained with Gel Red (Biotium, Fremont, CA, USA) against 1000 bp molecular mass marker (Sigma, Setagaya, Japan). Furthermore, appropriate bands were excised and purified using the Wizard^®^ SV Gel and PCR Clean-Up System (Promega, Madison, WI, USA), according to the manufacturer’s instructions. Purified amplicons were sequenced in Australian Genome Research Facility Ltd. (AGRF, Sydney node, NSW, Australia) using a Sanger dideoxy sequencer AB 3730xl (Applied Biosystems). Obtained raw data were trimmed, edited and deposited in GenBank (Table S1 available in the online version of this article). Furthermore, a total of 118 samples were subjected to qPCR assay to obtain the Ct values using the methods described previously [35].

For the NGS, DNA was isolated using the Qiagen Blood and Tissue mini kit (Qiagen, Hilden, Germany) as per the stated protocol [36,37]. The library was prepared using the Illumina DNA library preparation kit (Illumina, San Diego, CA, USA) and sequenced using the Illumina NextSeq sequencing platform, generating 150 bp paired-end reads. Sequencing data were analysed as per the established pipeline [36,38] using Geneious (version 10.2.2, Biomatters, Auckland, New Zealand) and CLC Genomics Workbench (version 9.5.4). Briefly, the raw reads were pre-processed to remove the Illumina adapter, ambiguous base calls and poor-quality reads (trim using quality score, limit 0.05; trim ambiguous nucleotide up to 15 using CLC Genomics Workbench), followed by mapping against the *Escherichia coli* bacterial genomic sequence (GenBank accession no. U00096) to remove possible bacterial contamination. Trimmed and unmapped clean reads were used as input data for de novo assembly in CLC Genomics Workbench (version 9.5.4). This resulted in the generation of a PiCV complete genome obtained from a Senegal dove (*Spilopelia senegalensis*) and PiCV partial Rep sequences from a plumed whistling duck (*Dendrocygna eytoni*).

### 2.3. Phylogenetic Reconstruction

The Bayesian phylogenetic tree was used to determine the genealogical relatedness of the PiCV with previously reported genotypes/strains in the NCBI database. Selected individual sequences were annotated with the accession number, country/region of circulation and collection year. A representative global alignment of the PiCV Rep gene (approximately 550 bp) from all full-length genomes was generated in Geneious with the MAFFT v7.017 (Research Institute for Microbial diseases, Osaka, Japan) alignment algorithm implemented in the Geneious package using the G-INS-i (gap open penalty 1.53; offset value 0.123). PiCV alignment jModelTest 2.1.3 favoured a general-time-reversible model with gamma distribution rate variation and a proportion of invariable sites (GTR + G + I) for phylogeny. A maximum clade credibility tree was inferred using the program Beast v1.10.4 [39]. In the MrBayes analysis, two independent Monte Carlo-Markov chains were implemented for 100 million generations each, with trees sampled every 5000 generations. The Bayesian skyline coalescent demographic prior was chosen because it allows for changes in population size over time [40]. Each analysis was checked to ensure that an appropriate effective sample size (ESS > 200) was achieved for all parameters. The final tree was visualised and edited in Fig tree 1.4 [41]. In addition, the avian host tree was pruned using the BirdTree service (https://birdtree.org; accessed on 12 November 2022) to generate the phylogeny subsets. During pruning, the Ericson tree of all species, a set of 10,000 trees, each with 9993 OTUs, was selected as the tree source to generate a set of 5000 trees [42]. The consensus tree was built with consensus Tree Builder using Geneious Prime software (V.2022.1.1), with 98% threshold support and 5% burn-in trees from a set of 5000 trees. Finally, the consensus tree was visualised and edited using FigTree V1.4.4 software. The tree was rooted at midpoint and the branches were transformed proportionally.

## 3. Results

### 3.1. PCR Detection of PiCV Infection

An approximately ~139 bp specific fragment was amplified using established PCR and qPCR. Later, another set of newly designed primers was used to amplify a ~515 bp Rep gene of the selected samples mentioned in Supplementary Table S2. The specific PCR products were identified as PiCV by sanger sequencing and BLAST analysis. Of the 82 samples from Australia, 60 were PiCV positive (positive rate, 60.98%). On the other hand, among 36 samples from Papua New Guinea, 8 were PiCV positive (positive rate, 22.22%) (details of the results of PCR and Ct values of qPCR can be seen in Supplementary Table S1 and Supplementary Table S3, respectively).

### 3.2. Bayesian Phylogeny of Extant PiCV in Australasia

**Figure 1:**
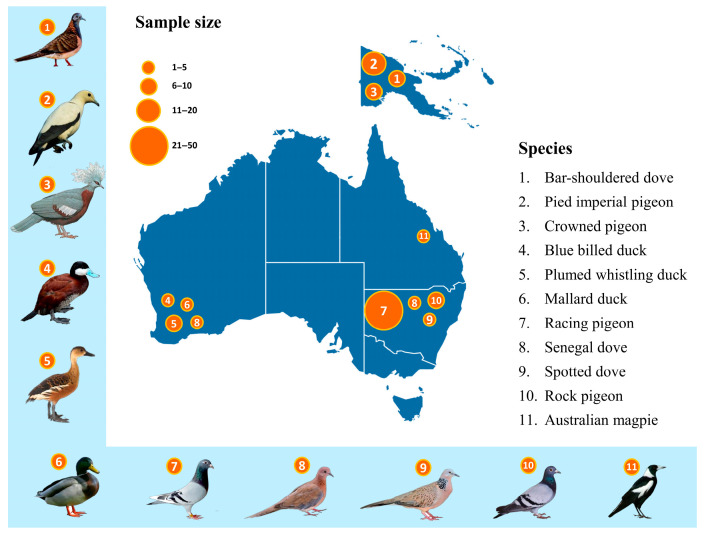
Location distribution in Australasia at which the described columbid species samples were obtained. Number of individuals and diversity of species at each site are illustrated and species’ names are depicted in the parenthesis.

A total of 14 new partial Rep sequences of PiCV were obtained from a spotted dove (*n* = 1), rock pigeons (*n* = 2), racing pigeons (*n* = 2), pied imperial pigeons (*n* = 3), a bar-shouldered dove (*n* = 1), a Scheepmaker’s crowned pigeon (*n* = 1), plumed whistling ducks (*n* = 2), a blue billed duck (*n* = 1) and a magpie (*n* = 1) from different geographic locations in Australia and deposited in GenBank (Accession nos. ON063537–ON063542, OM470912, OM470913, ON086796, ON086797) (Figure 1, Supplementary Table S2). In addition, one new complete genome sequence of PiCV was also attained from a Senegal dove from Perth, Western Australia and deposited in GenBank (Accession no. MZ447864) (Supplementary Table S2). Furthermore, four partial Rep sequences of PiCV (about 500 bp) were obtained from a spotted dove (*n* = 1), a rock pigeon (*n* = 1), a racing pigeon (*n* = 1) and a plumed whistling duck (*n* = 1) and deposited in GenBank (GenBank accession nos. OR587845–OR587848) (Supplementary Table S2). Eighteen new Australasian PiCV genomes showed >90% genome-wide pairwise similarity to one another, and >81% similarity to the 83 other complete PiCV genomes available in GenBank, sampled from various other parts of the world, including Poland, Australia, France, Italy, Northern Ireland, Belgium, China, Japan, USA and Germany.

**Figure 2:**
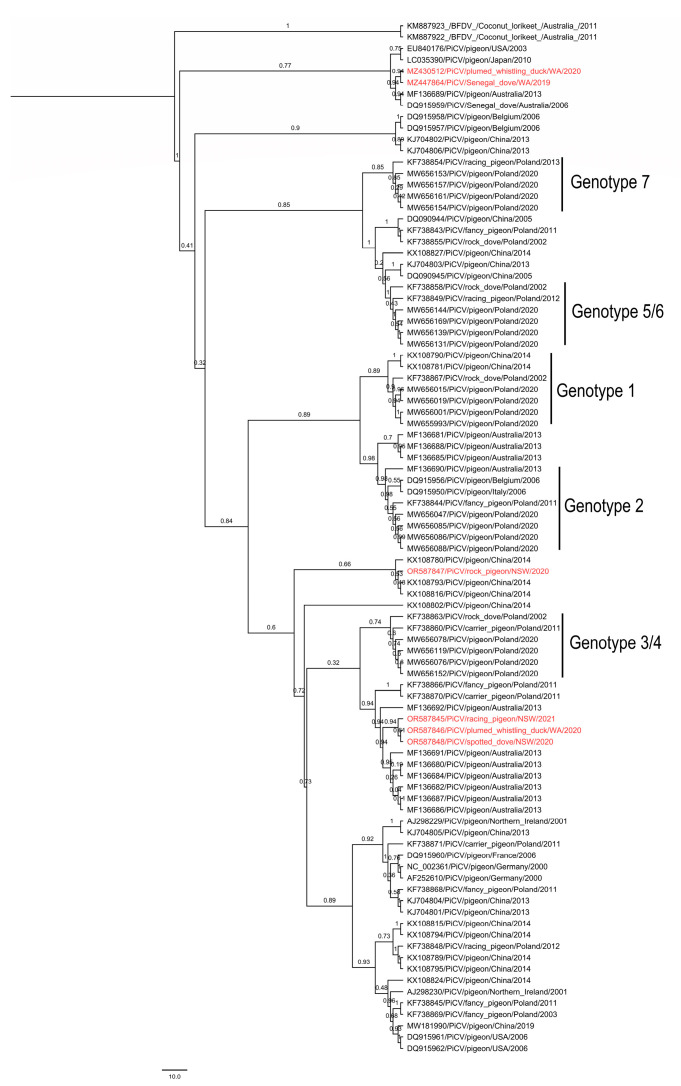
Bayesian phylogenetic inference of phylogenetic relationships amongst five partial *Rep* gene and one complete genome of PiCV isolated in the present study, with 83 others selected PiCV sequences available from NCBI. Clade posterior probability values are shown at tree nodes. Red taxa highlight the PiCV sequences isolated in this study. The isolates are marked as follows: accession number/virus name/host/country or region/year (with abbreviations WA: Western Australia; NSW: New South Wales). Genotypes were named as in previous study [43,44].

Global phylogenetic reconstruction was generated using a representative segment of Rep gene (approximately 550 bp) from the five partial Rep and one complete PiCV genome sequenced (GenBank accession nos. OR587845-OR587848; MZ447864) in this study, along with selected 83 PiCV genome sequences publicly available on NCBI. Two complete genomes of beak and feather disease virus (BFDV) from Coconut Lorikeet (GenBank accession nos. KM887922 and KM887923) were used as an outgroup during phylogenetic reconstruction. A Bayesian phylogenetic tree of Australian PiCV circulating in the reservoir, as well as aberrant hosts scattered across three different subclades (Figure 2). Firstly, one subclade comprised three PiCV genomes isolated from Australian racing pigeon, plumed whistling duck and spotted dove (GenBank accession nos. OR587845, OR587846 and OR587848), which demonstrated the strongest clade support of 94% with other Australian PiCV genomes (GenBank accession nos. MF136680, MF136682, MF136684, MF136686-87, MF136692) and spatially distant PiCV isolated from Poland (GenBank accession nos. KF738866 and KF738870). Secondly, a PiCV genome obtained from an Australian rock pigeon (GenBank accession no. OR587847) had medium clade (66%) support with a PiCV genome isolated from China (GenBank accession no. KX108793 and KX108816). Thirdly, a PiCV genome isolated from a Senegal dove (GenBank accession no. MZ447864) and a plumed whistling duck (GenBank accession no. MZ430512) had a strong clade support with a PiCV genome sequenced previously from a Senegal dove and an Australian feral pigeon in Australia, along with PiCV sequenced from USA and Japan (GenBank accession no. EU840176 and LC035390, respectively) (Figure 2).

### 3.3. PiCV Spillover Infection in Aberrant Hosts

Two plumed whistling ducks, one blue billed duck, one magpie and one Senegal dove were PCR positive for PiCV (Supplementary Table S2). Both the blood and feather sample of the plumed whistling ducks (CS 20-4427) were PCR positive, while in the blue billed duck (CS 20-3773), only the blood sample was PCR positive. In addition, the feather sample was PCR positive in both the magpie (CS 21-0553) and the Senegal dove (CS19-1715). The PCR results were verified at least twice during the initial testing period.

**Figure 3:**
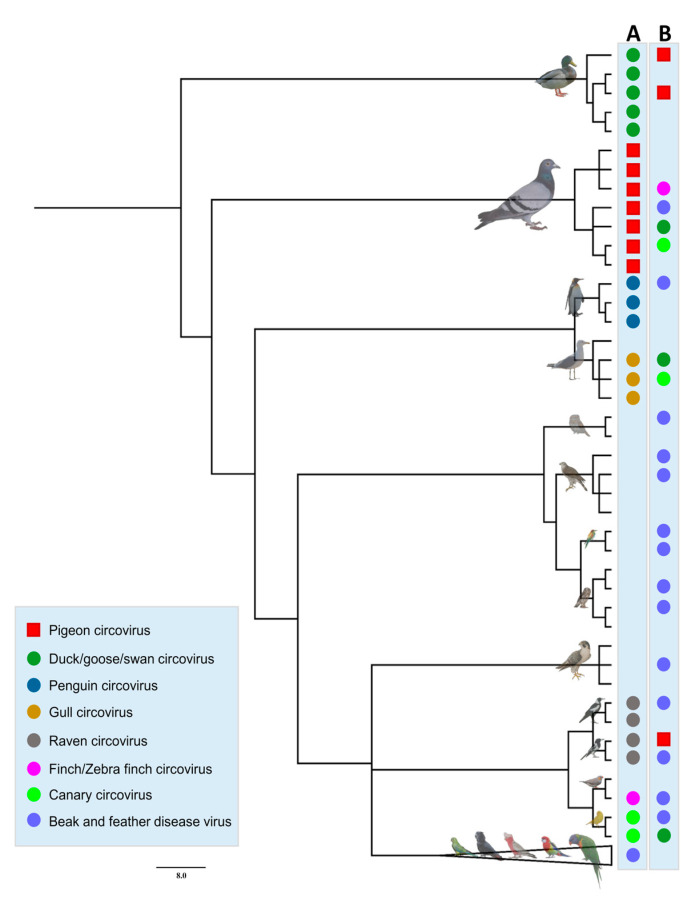
Patterns of avian circovirus spill over across the host phylogeny. Column A shows the extant circovirus in the respective hosts, where column B represents spillover of different circoviruses among the hosts. The node belonging to Psittaciformes hosts has been collapsed in the phylogenetic tree (see Figures S1 and S2 for original bootstraps support). The colored shapes in the tree representing different circoviruses are described in the legend.

**Figure 4:**
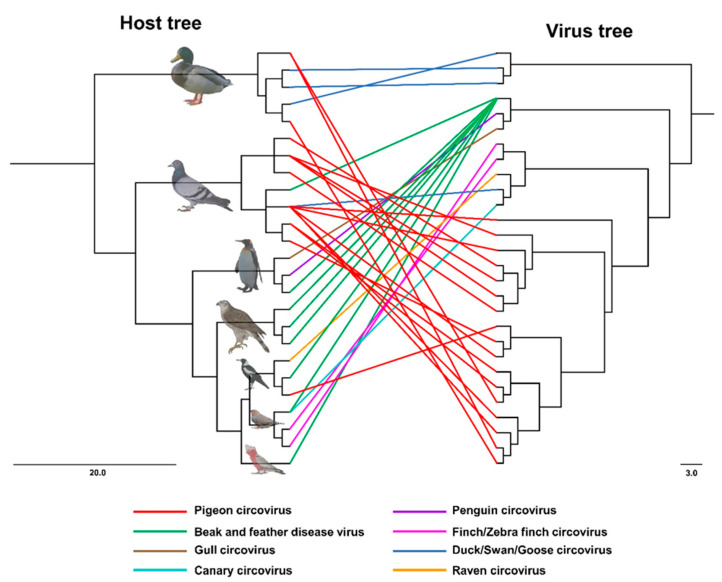
Tanglegram depicting the potential spillover events of circoviruses to different avian hosts. The host tree was pruned from the BirdTree service (https://birdtree.org) to generate the host phylogeny. Consensus tree was obtained using consensus Tree Builder using Geneious Prime software (V.2022.1.1) with 98% threshold support and 5% burn-in trees from a set of 5000 trees. Maximum likelihood trees were reconstructed from Rep sequences for circoviruses. Each tree was rooted at midpoint and the branches were proportionally transformed. Eight different colour indicates eight different avian circoviruses mentioned in the parenthesis.

Partial PiCV Rep sequences obtained from the plumed whistling duck (GenBank accession nos. MZ430510 and MZ430512), the blue billed duck (GenBank accession no. MZ430509) and the Australian magpie (GenBank accession no. MZ430511) represented inter-Order spillover of PiCV, even in the distantly related host, indicating the host generalist characteristics of the circovirus, whereas one entire genome of PiCV from the Senegal dove (GenBank accession no. MZ447864) and one partial Rep sequence of PiCV from the spotted dove (ON086796) displayed intra-Order spillover between closely related hosts (Figure 3). Moreover, the host virus co-phylogeny demonstrated potential spillover of the avian circoviruses in different hosts (Figure 4).

## 4. Discussion

Columbiformes are a globally distributed group of birds, with Australasia being home to a significant proportion of the world’s columbid fauna, including 22 species [45,46]. Several studies have been conducted on PiCV in domestic and feral pigeons across the world to draw a plausible phylogenetic relationship among the existing genotypes [17,19,47,48,49]. These phylogenetic and recombination analyses demonstrated that PiCV obtained from rock doves is genetically highly diverse and prone to genetic recombination. Circoviruses are widespread infections across the tree of life, occupying host niches over millennia with high evolutionary speed [31] and genetic recycling through recombination. Host-specific sympatric speciation has already been discovered in another avian circovirus like BFDV in Australia [31]. Therefore, it can be expected that the genetic population structure of PiCV might follow a similar trajectory as BFDV in parrots and cockatoos. Like the Psittaciformes, the Order Columbiformes includes a diverse group of birds with 310 recognized species around the world, with the greatest diversity present in Australasia [19]. In this context, our present study was conducted to better understand the genetic diversity of PiCV in a variety of avian hosts from different geographical locations of Australasia. PiCV from diverse range of hosts were screened in various geographical locations of Australasia, including Papua New Guinea (Figure 1). The PiCV sequences obtained from reservoir and aberrant hosts showed >90% genome-wide pairwise similarity to one another, and >81% similarity to all the 83 other complete PiCV genomes available in GenBank. Global phylogenetic reconstruction of PiCV rep sequences obtained in the present study with selected PiCV complete genomes publicly available on GenBank demonstrated the absence of a consistent genetic population structure (Figure 2). The PiCV sequences identified in different avian hosts are distributed throughout the phylogenetic tree, clearly highlighting the lack of a host-adapted population structure of diverse PiCVs circulating in Australasia. This is possibly due to the small number of sequences from diverse species of birds and the lack of a complete PiCV genome from the present study. A phylogenetic analysis based on the complete genome of the PiCV from diverse pigeon species would be essential to present real genetic diversity. In the present study, five partial rep sequences and one full genome of PiCV were used to draw a genealogical relatedness of the PiCV. However, partial Rep sequences obtained from a plumed whistling duck, a blue billed duck, a spotted dove, a magpie and a Senegal dove from Australia and a pied imperial pigeon, a bar-shouldered dove and a Scheepmaker’s pigeon from Papua New Guinea (PNG) indicate the spillover infection in the aberrant host. Dried blood spots were used for DNA extraction to conduct PCR and sequencing. It is plausible that no replicating virus was present in the blood samples used in the present study. Therefore, it was difficult to obtain the full genome (FG) from the dried blood spots stored in our laboratory for a long time. Amplification of the FG of the virus using the RCA (Rolling Circle Amplification) method failed in our laboratory. It might be the results of DNA fragmentation during preservation of dried blood spot on the FTA card.

Global phylogenetic analyses demonstrated that the PiCV genomes circulating in Australia are scattered across three different subclades, which likely indicates that these pigeons are closely related with each other in terms of genetic similarity. *Columba livia domestica* is known for its cosmopolitan distribution and bred for different purposes such as sporting/racing, fancy and utility, et cetera. As sport and race are the most popular reasons for pigeon breeding and farming, this allows for the close contact of pigeons from different domestic and even international lofts during transport. Therefore, pigeon circovirus has also been detected in many countries across the world. Sporting events also provide opportunities for interacting among feral and racing pigeons. Additionally, international trade for pigeons with good pedigrees to be used as breeding stock has also been suggested to facilitate the international distribution of this virus, leading to the formation of different genotypic clades among the PiCV [10,15]. A study of PiCV complete genomes from the pigeons of PNG is likely to be essential to establish a clear evolutionary relationship of PiCV in Columbiformes.

In previous phylogenetic studies, five genotypic clades, termed A to E, were identified based on differences in the sequence of the PiCV genome. While clade A included European, American and Asian isolates, clade B, C and D consists of European, South American and Asian isolates. Clade E is composed of the most genetically distant strains isolated from the domestic pigeon in the USA, the Senegal dove (*Streptopelia senegalensis*) in Australia and the racing pigeon in Japan [18,48,49,50]. Although several studies showed that PiCV clustered into different genotypes, there was no obvious relationship between clades and geographical origin [10,16,43]. However, we observed in the phylogenetic tree the tendency to group sequences from individual studies, which might indicate clustering of PiCV sequence based on temporality, country of isolation or an interface of both (Figure 2). These observations apparently correspond with recent findings [44]. On the other hand, a Bayesian phylogenetic tree of PiCV genomes sequenced in Australia formed three strongly supported monophyletic clades. Whereas one clade was clustered with PiCV isolated from Poland and Belgium, the second clade formed a geographical clustering with other PiCV isolated globally, indicating susceptibility to different genotypes. A third clade produced a cluster with the only other Australian PiCV genome (DQ915959) obtained from a feral Senegal dove (*Streptopelia senegalensis*) in Western Australia [19]. Recent phylogenetic studies based on cap and full genomes demonstrated that PiCV strains could be further divided into seven clades, and some of the PiCV sequences are similar to worldwide strains from different types of pigeons [17,43]. Present phylogenetic analyses demonstrated that PiCV sequences from the same geographical location of Australia were distributed throughout the phylogenetic tree. Although complete genomes of PiCV from ten different countries were included in this analyses, more samples of whole PiCV genomes are encouraged to be sequenced, especially from understudied regions and/or countries in order to come up with a better resolution of the phylogenetic association.

PiCV has a broad host range and is widespread in Columba livia, but in several cases, PiCV genetic material was also detected in the Senegal dove (*Streptopelia senegalensis*) and the collared dove (*Streptopelia decaocto*) [22,51]. In a recent study, partial or complete PiCV sequences were obtained from 15 avian hosts and one from a parasitic arachnid [44]. Natural spillover infection of PiCV into distantly related hosts, such as the plumed whistling duck, the blue billed duck and the Australian magpie, has been documented in the present study. PiCV DNA was also detected from the Senegal dove, the pied imperial pigeon bar-shouldered dove, Scheepmaker’s crowned pigeon and the spotted dove, which broadens the natural range of hosts. Adaptation of the cryptic host is postulated to be the major driver for the establishment of new BFDV infections in the orange bellied parrot [32]. Analysis of host-virus cophylogeny highlighted the competing forces of co-divergence and cross-species transmissions in the current distribution of the BFDV genetic population [32]. Australasia is a rich source of biogeographical diversity of avian host species including the Passeriformes, Psittaciformes, Columbiformes and Anseriformes, which are natural hosts of avian circovirus species [19]. Like other circovirus species, PiCV is highly diverse and prone to genetic recombination [10,17,19,48], which could be the potential source of cross species transmission. Wild pigeons might act as the reservoirs of multiple viral pathogens for cross-species transmission [19,52]. Regardless of the PiCV sequence obtained in the present study, it is very difficult to explain the case without conducting ethically debatable experimental virus-transmission experiments. As ducks and magpies do not intimately share any ecological niche with pigeons, the PiCV genotypes detected currently infecting ducks are likely the result of spillover through contact with doves or pigeons on zoo premises, as the species is known to forage in mixed flocks. Circovirus is profusely shed in the faeces and feather dust of infected birds [6,8], with feather dander from circovirus-infected birds containing as many as one billion virus particles per microlitre [6]. Transmission occurs through direct contact, ingestion, inhalation of contaminated aerosols or via infected fomites [53]. Like other circoviruses, PiCV is resilient and can readily withstand temperatures of 80–85 °C, which may lead to long-term contamination of the dove cote, possibly for many years. The oral or cloacal transmission and environmental persistence of PiCV, as well as the close contact between ducks and pigeons on the premises facilitating spillover, allows pigeons to act as reservoirs for circovirus infection in domestic and wild ducks. Absence of a complete genome of PiCV for phylogenetic reconstruction, opportunistic sampling and small numbers of pigeon samples from the various geographical locations of Australia are the limitations of our study. In the future, more aberrant hosts will be tested for detection of PiCV spillover infection in Australia. Moreover, a greater number of PiCV from Australasia will be sequenced to better understand the phylogeographic niches of PiCV in pigeons.

## 5. Conclusions

This study provided an excellent opportunity to reveal the genealogical relationship of PiCV among diverse species of pigeons widespread in Australasia, highlighting the host generalist characteristics of PiCV. These outcomes would facilitate the control and prevention of PiCV infections in domestic and wild pigeons in Australia.

## Data Availability

Viral sequences were submitted to GenBank^®^ and accession number(s) can be found in the article/Supplementary material.

## Supplementary Materials

The following supporting information can be downloaded at: https://www.mdpi.com/article/10.3390/v15102025/s1, Figure S1: Avian host tree pruned from the BirdTree service (https://birdtree.org) to generate the host phylogeny. Figure S2: Avian host tree pruned from the BirdTree service (https://birdtree.org) to generate the host phylogeny. Table S1: PCR detection of PiCV in Australia and Papua New Guinea. Table S2: PiCV partial Rep sequences obtained from the present study. Complete genome of PiCV found in this study is marked in bold. Table S3: Ct value of DNA samples tested for PiCV by using qPCR.
