# Supplementary material for: Australasian Pigeon Circoviruses Demonstrate Natural Spillover Infection
Source: Viruses. 2023 Sep 29;15(10):2025. doi: 10.3390/v15102025 (PMC10611180; doi:10.3390/v15102025)
Supplement: Supplementary file 1 [file viruses-15-02025-s001.zip › viruses-2573213-supplementary.pdf]

Supplementary Materials

Australasian Pigeon Circoviruses Demonstrate Natural Spillover Infection

*** Correspondence:** bnath@csu.edu.au

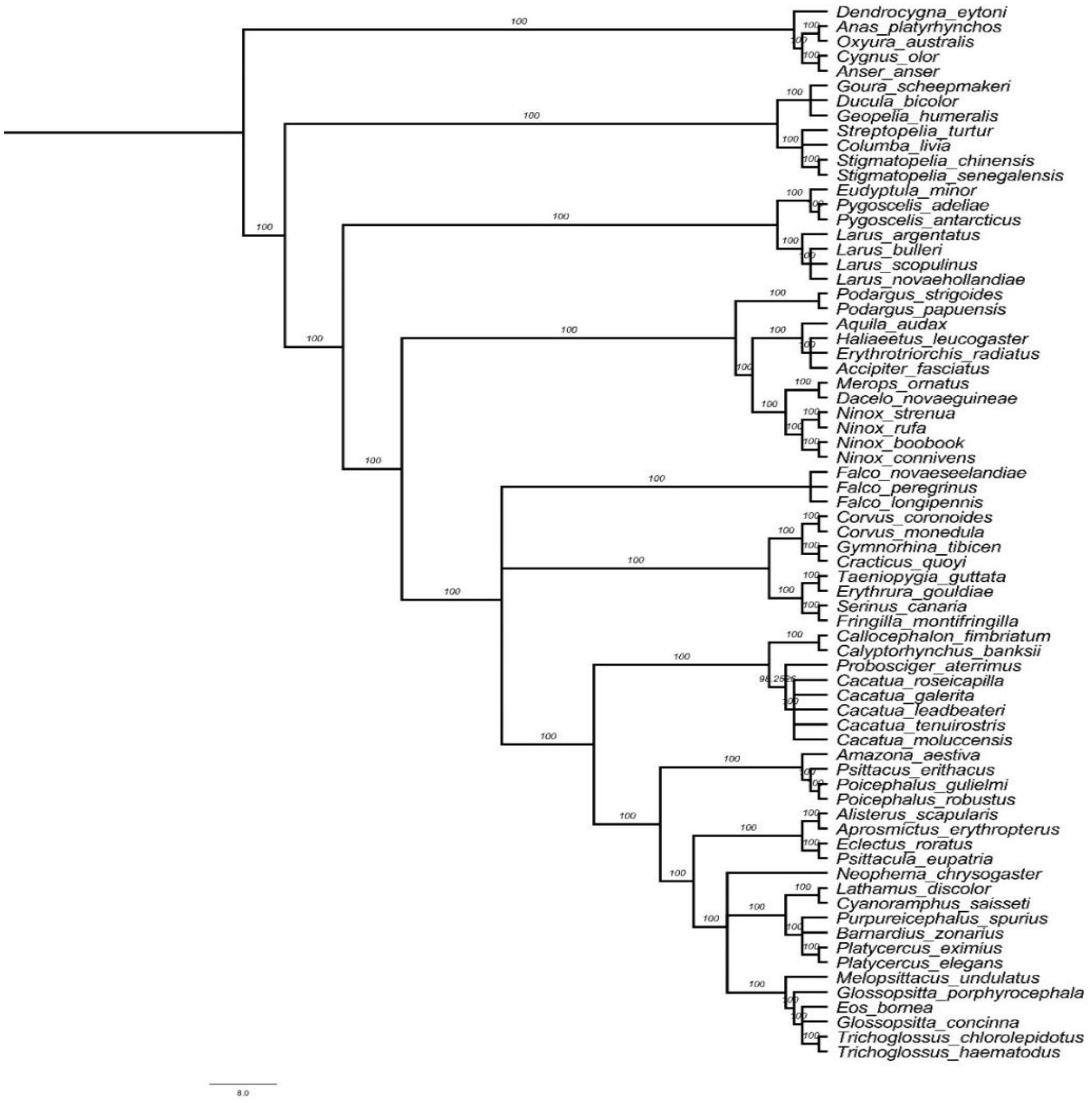

Figure S1. Avian host tree pruned from the BirdTree service (https://birdtree.org) to generate the host phylogeny. Consensus tree was obtained using consensus Tree Builder using Geneious Prime software (V.2022.1.1) with 98% Threshold support and 5% burn-in trees from a set of 5000 trees. The tree was rooted at midpoint and the branches were proportionally transformed. Consensus support for each branch was displayed as percent value (%) over the branch.

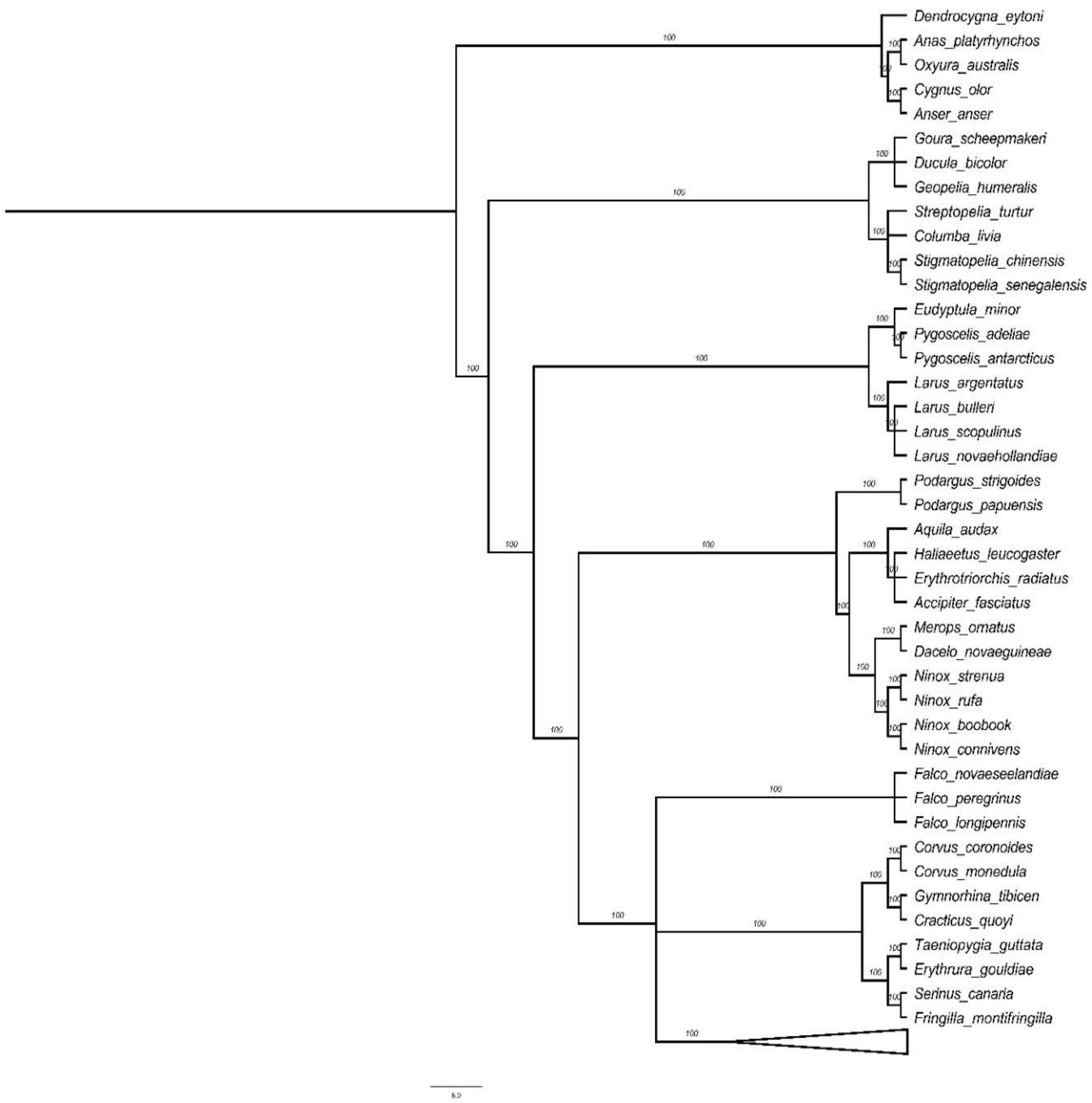

Figure S2. Avian host tree pruned from the BirdTree service (https://birdtree.org) to generate the host phylogeny. Consensus tree was obtained using consensus Tree Builder using Geneious Prime software (V.2022.1.1) with 98% Threshold support and 5% burn-in trees from a set of 5000 trees. The tree was rooted at midpoint and the branches were proportionally transformed. Consensus support for each branch was displayed as percent value (%) over the branch. The node belongs to Psittaciformes hosts has been collapsed in this tree.

Table S1: PCR detection of PiCV in Australia and Papua New Guinea

Host	Species	Australia		PNG	
		**Bird Tested**	**Positive**	**Bird Tested**	**Positive**
Racing Pigeon	*Columba livia*	48	38	0	0
Pied imperial pigeon	*Ducula bicolor*	0	0	18	4
Bar-shouldered dove	*Geopelia humeralis*	0	0	10	2
Scheepmaker's pigeon	*Goura scheepmakeri*	0	0	8	2
Senegal dove	*Streptopelia senegalensis*	2	1	0	0
Spotted dove	*Streptopelia chinensis*	2	1	0	0
Rock pigeon	*Columba livia*	10	4	0	0
Plumed whistling duck	*Dendrocygna eytoni*	6	2	0	0
Blue billed duck	*Oxyura australis*	5	2	0	0
Mallard duck	*Anas platyrhynchos*	5	0	0	0
Australian magpie	*Gymnorhina tibicen*	4	2	0	0
Total =		82	50 (60.98%)	36	8 (22.22%)

Table S2: PiCV partial *Rep* sequences obtained from the present study. Complete genome of PiCV found in this study is marked in bold.

GenBank accession no.	Year	Isolate	Location	Species	Source of samples	Size of Sequences (bp)
OM470912	2021	CS21-0422	NSW	*Columba livia*	Liver and spleen	111
OM470913	2021	CS21-0422	NSW	*Columba livia*	Oropharynx	111
ON063538	2013	VDL-1	PNG	*Ducula bicolor*	Blood	113
ON063539	2013	VDL-2	PNG	*Ducula bicolor*	Blood	113
ON063540	2013	VDL-3	PNG	*Ducula bicolor*	Blood	113
ON063541	2013	VDL-4	PNG	*Geopelia humeralis*	Blood	113
ON063542	2013	VDL-5	PNG	*Goura scheepmakeri*	Blood	113
**MZ447864**	**2019**	**CS19-1715**	**WA**	***Streptopelia senegalensis***	**Liver**	**2039**
ON086796	2020	CS20-0321	NSW	*Streptopelia chinensis*	bursa of Fabricius	110
ON086797	2020	CS20-1270	NSW	*Columba livia*	Blood	110
ON063537	2020	CS20-1270	NSW	*Columba livia*	Liver	110
MZ430510	2020	CS20-4427	WA	*Dendrocygna eytoni*	Blood	110
MZ430512	2020	CS20-4427	WA	*Dendrocygna eytoni*	Feather	699
MZ430509	2020	CS20-3773	WA	*Oxyura australis*	Blood	110
MZ430511	2021	CS21-0553	QLD	*Gymnorhina tibicen*	Feather	112
OR587845	2021	CS21-0422	NSW	*Columba livia*	Liver	432
OR587846	2020	CS20-4427	WA	*Dendrocygna eytoni*	Feather	502
OR587847	2020	CS20-1270	NSW	*Columba livia*	Blood	458
OR587848	2020	CS20-0321	NSW	*Streptopelia chinensis*	bursa of Fabricius	465

Table S3: Ct value of DNA samples tested for PiCV qPCR.

Sl. No.	VDL ID	Sample type	Host	PCR (P/N)	Ct value (qPCR)
ID-1	CS20-4427_1	Blood	Plumed whistling duck	P	38.74
ID-2	CS20-4427_2	Feather	Plumed whistling duck	P	30.74
ID-3	CS20-4427_3	Feather	Plumed whistling duck	N	ND
ID-4	CS20-4427_4	Blood	Plumed whistling duck	N	ND
ID-5	CS20-4427_5	Feather	Plumed whistling duck	N	ND
ID-6	CS20-4427_6	Feather	Plumed whistling duck	N	ND
ID-7	CS20-3773_1	Blood	Blue Billed Duck	P	31.13
ID-8	CS20-3773_2	Feather	Blue Billed Duck	P	31.1
ID-9	CS20-3773_3	Feather	Blue Billed Duck	N	ND
ID-10	CS20-3773_4	Blood	Blue Billed Duck	N	ND
ID-11	CS20-3773_5	Feather	Blue Billed Duck	N	ND
ID-12	CS21-0509_1	Feather	Mallard Duck	N	ND
ID-13	CS21-0509_2	Feather	Mallard Duck	N	ND
ID-14	CS21-0509_3	Feather	Mallard Duck	N	ND
ID-15	CS21-0509_4	Feather	Mallard Duck	N	ND
ID-16	CS21-0509_5	Feather	Mallard Duck	N	ND
ID-17	CS20-0321_1	bursa of Fabricius	Spotted dove	P	30.59
ID-18	CS20-0321_2	Feather	Spotted dove	N	ND
ID-19	CS21-0553_1	Feather	Australian magpie	P	30.57
ID-20	CS21-0553_2	Feather	Australian magpie	P	31.22
ID-21	CS21-0553_3	Feather	Australian magpie	N	ND
ID-22	CS21-0553_4	Feather	Australian magpie	N	ND
ID-23	CS20-1270_1	Liver	Rock Pigeon	P	16.76
ID-24	CS20-1270_2	Lung	Rock Pigeon	P	15.83
ID-25	CS20-1270_3	Spleen	Rock Pigeon	P	29.46
ID-26	CS20-1270_4	Blood	Rock Pigeon	P	18.66
ID-27	CS20-1270_5	Blood	Rock Pigeon	N	ND
ID-28	CS20-1270_6	Blood	Rock Pigeon	N	ND
ID-29	CS20_4890_1	Cloacal swab	Rock Pigeon	N	ND
ID-30	CS20_4890_2	Cloacal swab	Rock Pigeon	N	ND
ID-31	CS20_4890_3	Cloacal swab	Rock Pigeon	N	ND
ID-32	CS20_4890_4	Cloacal swab	Rock Pigeon	N	ND
ID-33	CS19-1715_1	Liver	Senegal dove	P	18.78
ID-34	CS19-1715_2	Feather	Senegal dove	N	ND
ID-35	N/A	Blood Spot	Pied imperial pigeon	P	32.98
ID-36	N/A	Blood Spot	Pied imperial pigeon	N	ND
ID-37	N/A	Blood Spot	Pied imperial pigeon	N	ND
ID-38	N/A	Blood Spot	Pied imperial pigeon	N	ND
ID-39	N/A	Blood Spot	Pied imperial pigeon	N	ND
ID-40	N/A	Blood Spot	Pied imperial pigeon	N	ND
ID-41	N/A	Blood Spot	Pied imperial pigeon	P	32.01

ID-42	N/A	Blood Spot	Pied imperial pigeon	N	ND
ID-43	N/A	Blood Spot	Pied imperial pigeon	N	ND
ID-44	N/A	Blood Spot	Pied imperial pigeon	P	29.39
ID-45	N/A	Blood Spot	Pied imperial pigeon	N	ND
ID-46	N/A	Blood Spot	Pied imperial pigeon	N	ND
ID-47	N/A	Blood Spot	Pied imperial pigeon	N	ND
ID-48	N/A	Blood Spot	Pied imperial pigeon	N	ND
ID-49	N/A	Blood Spot	Pied imperial pigeon	P	31.42
ID-50	N/A	Blood Spot	Pied imperial pigeon	N	ND
ID-51	N/A	Blood Spot	Pied imperial pigeon	N	ND
ID-52	N/A	Blood Spot	Pied imperial pigeon	N	ND
ID-53	N/A	Blood Spot	Bar-shouldered dove	P	30.88
ID-54	N/A	Blood Spot	Bar-shouldered dove	N	ND
ID-55	N/A	Blood Spot	Bar-shouldered dove	N	ND
ID-56	N/A	Blood Spot	Bar-shouldered dove	N	ND
ID-57	N/A	Blood Spot	Bar-shouldered dove	N	ND
ID-58	N/A	Blood Spot	Bar-shouldered dove	P	31.44
ID-59	N/A	Blood Spot	Bar-shouldered dove	N	ND
ID-60	N/A	Blood Spot	Bar-shouldered dove	N	ND
ID-61	N/A	Blood Spot	Bar-shouldered dove	N	ND
ID-62	N/A	Blood Spot	Bar-shouldered dove	N	ND
ID-63	N/A	Blood Spot	Scheepmaker's pigeon	P	31.68
ID-64	N/A	Blood Spot	Scheepmaker's pigeon	N	ND
ID-65	N/A	Blood Spot	Scheepmaker's pigeon	N	ND
ID-66	N/A	Blood Spot	Scheepmaker's pigeon	N	ND
ID-67	N/A	Blood Spot	Scheepmaker's pigeon	N	ND
ID-68	N/A	Blood Spot	Scheepmaker's pigeon	P	30.86
ID-69	N/A	Blood Spot	Scheepmaker's pigeon	N	ND
ID-70	N/A	Cloacal swab	Scheepmaker's pigeon	N	ND
ID-71	CS 21-0422_1	Liver	Racing pigeon	P	15.22
ID-72	CS 21-0422_2	Trachea	Racing pigeon	P	17.85
ID-73	CS 21-0422_3	bursa of Fabricius	Racing pigeon	P	16.38
ID-74	CS 21-0422_4	Oropharynx	Racing pigeon	P	12.58
ID-75	CS 21-0422_5	Oro-cloacal swab	Racing pigeon	N	33.62
ID-76	CS 21-0422_6	Oro-cloacal swab	Racing pigeon	P	27.46
ID-77	CS 21-0422_7	Oro-cloacal swab	Racing pigeon	P	31.02
ID-78	CS 21-0422_8	Oro-cloacal swab	Racing pigeon	P	28.6
ID-79	CS 21-0422_9	Oro-cloacal swab	Racing pigeon	P	29.92
ID-80	CS 21-0422_10	Oro-cloacal swab	Racing pigeon	N	33.01
ID-81	CS 21-0422_11	Oro-cloacal swab	Racing pigeon	P	27.25
ID-82	CS 21-0422_12	Oro-cloacal swab	Racing pigeon	P	31.55
ID-83	CS 21-0422_13	Oro-cloacal swab	Racing pigeon	P	31.07
ID-84	CS 21-0422_14	Oro-cloacal swab	Racing pigeon	P	29.07
ID-85	CS 21-0422_15	Oro-cloacal swab	Racing pigeon	N	32.19

ID-86	CS 21-0422_16	Oro-cloacal swab	Racing pigeon	P	28.64
ID-87	CS 21-0422_17	Oro-cloacal swab	Racing pigeon	N	31.64
ID-88	CS 21-0422_18	Oro-cloacal swab	Racing pigeon	P	31.4
ID-89	CS 21-0422_19	Oro-cloacal swab	Racing pigeon	P	30.83
ID-90	CS 21-0422_20	Oro-cloacal swab	Racing pigeon	P	29.06
ID-91	CS 21-0422_21	Oro-cloacal swab	Racing pigeon	N	32.29
ID-92	CS 21-0422_22	Oro-cloacal swab	Racing pigeon	P	30.91
ID-93	CS 21-0422_23	Oro-cloacal swab	Racing pigeon	P	31.21
ID-94	CS 21-0422_24	Oro-cloacal swab	Racing pigeon	P	29.53
ID-95	CS 21-0422_25	Oro-cloacal swab	Racing pigeon	N	31.99
ID-96	CS 21-0422_26	Oro-cloacal swab	Racing pigeon	P	31.58
ID-97	CS 21-0422_27	Oro-cloacal swab	Racing pigeon	P	18.2
ID-98	CS 21-0422_28	Oro-cloacal swab	Racing pigeon	P	27.5
ID-99	CS 21-0422_29	Oro-cloacal swab	Racing pigeon	P	14.46
ID-100	CS 21-0422_30	Oro-cloacal swab	Racing pigeon	N	38.71
ID-101	CS 21-0422_31	Spleen	Racing pigeon	P	13.27
ID-102	CS 21-0422_32	Lung	Racing pigeon	P	21.97
ID-103	CS 21-0422_33	Lymph node	Racing pigeon	P	15.65
ID-104	CS 21-0422_34	Oro-cloacal swab	Racing pigeon	P	30
ID-105	CS 21-0422_35	Oro-cloacal swab	Racing pigeon	P	22.73
ID-106	CS 21-0422_36	bursa of Fabricius	Racing pigeon	P	16.6
ID-107	CS 21-0422_37	Oro-cloacal swab	Racing pigeon	P	28.88
ID-108	CS 21-0422_38	bursa of Fabricius	Racing pigeon	P	19.16
ID-109	CS 21-0422_39	Oro-cloacal swab	Racing pigeon	P	27.54
ID-110	CS 21-0422_40	Oro-cloacal swab	Racing pigeon	P	25.25
ID-111	CS 21-0422_41	Oro-cloacal swab	Racing pigeon	P	24.79
ID-112	CS 21-0422_42	Oro-cloacal swab	Racing pigeon	P	27.18
ID-113	CS 21-0422_43	Oro-cloacal swab	Racing pigeon	N	38.01
ID-114	CS 21-0422_44	Oro-cloacal swab	Racing pigeon	P	18.2
ID-115	CS 21-0422_45	Oro-cloacal swab	Racing pigeon	N	33.19
ID-116	CS 21-0422_46	Oro-cloacal swab	Racing pigeon	N	33.88
ID-117	CS 21-0422_47	Oro-cloacal swab	Racing pigeon	P	31.29
ID-118	CS 21-0422_48	Oro-cloacal swab	Racing pigeon	P	30.67

“P” denotes "Postive" and “N” denotes "Negative"; “ND” denotes “Not detected”; “N/A” denotes “Not applicable”
